# Dosage of the pseudoautosomal gene *SLC25A6* is implicated in QTc interval duration

**DOI:** 10.1038/s41598-023-38867-3

**Published:** 2023-07-26

**Authors:** Anne Skakkebæk, Kasper Kjær-Sørensen, Vladimir V. Matchkov, Lise-Lotte Christensen, Jesper Just, Cagla Cömert, Niels Holmark Andersen, Claus Oxvig, Claus Højbjerg Gravholt

**Affiliations:** 1 Department of Clinical Genetics, Aarhus University Hospital, Palle Juul-Jensens Boulevard 99, 8200 Aarhus N, Denmark; 2 Department of Molecular Medicine, Aarhus University Hospital, Aarhus, Denmark; 3 Department of Clinical Medicine, Aarhus University Hospital, Aarhus, Denmark; 4 Department of Molecular Biology and Genetics, Aarhus University, Aarhus, Denmark; 5 Department of Biomedicine, Aarhus University, Aarhus, Denmark; 6 Research Unit for Molecular Medicine, Department of Clinical Medicine, Aarhus University and Aarhus University Hospital, Aarhus, Denmark; 7 Department of Cardiology, Aalborg University Hospital, Aalborg, Denmark; 8 Department of Endocrinology and Internal Medicine and Medical Research Laboratories, Aarhus University Hospital, Aarhus, Denmark

**Keywords:** Clinical genetics, Gene expression, Sequencing, Cardiology, Diseases, Molecular medicine

## Abstract

The genetic architecture of the QT interval, defined as the period from onset of depolarisation to completion of repolarisation of the ventricular myocardium, is incompletely understood. Only a minor part of the QT interval variation in the general population has been linked to autosomal variant loci. Altered X chromosome dosage in humans, as seen in sex chromosome aneuploidies such as Turner syndrome (TS) and Klinefelter syndrome (KS), is associated with altered QTc interval (heart rate corrected QT), indicating that genes, located in the pseudoautosomal region 1 of the X and Y chromosomes may contribute to QT interval variation. We investigate the dosage effect of the pseudoautosomal gene *SLC25A6,* encoding the membrane ADP/ATP translocase 3 in the inner mitochondrial membrane, on QTc interval duration. To this end we used human participants and in vivo zebrafish models. Analyses in humans, based on 44 patients with KS, 44 patients with TS, 59 male and 22 females, revealed a significant negative correlation between *SLC25A6* expression level and QTc interval duration. Similarly, downregulation of *slc25a6* in zebrafish increased QTc interval duration with pharmacological inhibition of K_ATP_ channels restoring the systolic duration, whereas overexpression of *SLC25A6* shortened QTc, which was normalized by pharmacological activation of K_ATP_ channels. Our study demonstrate an inverse relationship between *SLC25A6* dosage and QTc interval indicating that *SLC25A6* contributes to QT interval variation.

## Introduction

The electrocardiographic QT interval reflects depolarization and repolarization of the myocardium, and patients with an either prolonged or shortened QT interval are at risk of arrhythmias and sudden cardiac death^1^. The pathophysiology and genetic architecture of the QT interval is incompletely understood. Pathogenic variants in several autosomal genes encoding cardiac potassium channels have been identified as implicated in Mendelian inherited long QT syndrome and short QT syndrome^2^. However, only a minor part of the QT interval variation in the general population has been linked to common autosomal variant loci through genome-wide association studies^3–5^, leaving a major part of the QT interval variation genetically elusive.

In the early twentieth century, Henry Cuthbert Bazett demonstrated that the QT interval duration displayed sexual dimorphism, with women having a 6% longer average frequency corrected QT (QTc) interval than men^6^. This sexual dimorphism is believed to be driven by sex hormones^7^. Recently, extensive sex differences at the transcription level of both autosomal and sex chromosomal genes have been identified^8,9^ suggesting an impact of sex chromosome dosage in sexual dimorphism in health and diseases.

Sex chromosome aneuploidies (SCAs) constitute a valuable human model to advance knowledge of sex chromosome dosage in health and disease. Interestingly, studies of the QTc interval in patients with Turner syndrome (TS; 45,X) and Klinefelter syndrome (KS; 47,XXY) have revealed that the QTc interval is longer in patients with TS^10–12^, while patients with KS have a shortened QTc interval^13,14^. Notably, in a study of patients with KS, QTc interval was found to be significantly shorter in patients expressing a higher level of autosomal genes *DOCK7* and *GSTM2*, the X chromosomal gene SMCA1, as well as pseudoautosomal genes *CD99*, *SLC25A6*, *P2RY8*, *ZBED1*, and *GTPBP6*^14^. *SLC25A6*, Solute Carrier Family 25 Member 6, encodes the membrane ADP/ATP translocase 3, a membrane protein located in the inner mitochondrial membrane, which is involved in the exchange of intra-mitochondrial ATP for cytoplasmic ADP^15^, plays a key role in repiratory ATP synthesis, which may be critical for QTc duration. *SLC25A6* is expressed in various levels in almost all human tissues, including cardiomyocytes^16^. Single cell transcriptomics of human heart tissue futher revealed that *SLC25A6* is expressed in all cell types, including cardiomyocytes and smooth muscle cells (www.proteinatlas.org)^17^ (see Supplementary Fig. 1). Compared to female and male controls, TS patients show lower expression^18,19^, while patients with KS show higher expression of *SLC25A6*^14,18–21^.

To determine a possible effect of altered dosage of *SLC25A6* on the duration of the QT interval, we used both human participants including patients with TS and KS and zebrafish models. The zebrafish has been emphasized as a successful model to investigate the role of specific genes in human diseases and development^22^ and specifically in heart physiology^23,24^. Although the heart of zebrafish, like the heart from any common model organism, differs from the human heart, action potential in zebrafish cardiomyocytes shares the main characteristics of action potential of human ventricle myocytes^25^. The ionic current background of membrane potential changes in zebrafish cardiomyocytes differs slightly from humans, but the majority of ion channel orthologs important for human ventricular cardiomyocyte action potential are expressed in zebrafish heart. These include the sodium and calcium voltage-gated channels, the inward rectifying and ATP-dependent potassium channels. Therefore, cardiomyocyte action potential shape and duration result in heart rate-dependent QT-interval, which is highly similar between zebrafish and humans, making the zebrafish an attractive model organism for studying the effect of aberrant gene expression on cardiac physiology of relevance to humans^23,24^. The zebrafish genome contains a single highly conserved *SLC25A6* orthologue, *slc25a6*, encoding a protein with 92% sequence identity to human *SLC25A6*. Thus, the zebrafish presents an attractive model to assess the consequences of altered *SLC25A6* expression on cardiac rhythmicity in vivo.

In our case–control study, including TS, KS and female and male controls, we showed a significant negative correlation between the expression level of *SLC25A6* and QTc interval duration. Similarly, in zebrafish, we observed that downregulation of *slc25a6* resulted in a prolongation of the QTc interval whereas overexpression of *SLC25A6* resulted in a shortening of the QTc interval. As a therapeutic intervention, we investigated the effect of pharmacological K_ATP_ channel inhibition in zebrafish with downregulated *slc25a6* and K_ATP_ channel opening in zebrafish with overexpression of SLC25A6. Both interventions rescued the phenotypes. Collectively, our data shows that dosage of *SLC25A6* impacts QTc interval and that these changes can bepharmacologically reversible.

## Results

### *SLC25A6* expression levels correlate with sex-chromosome dosage in humans across tissue type of mesoderm origin

**Figure 1:**
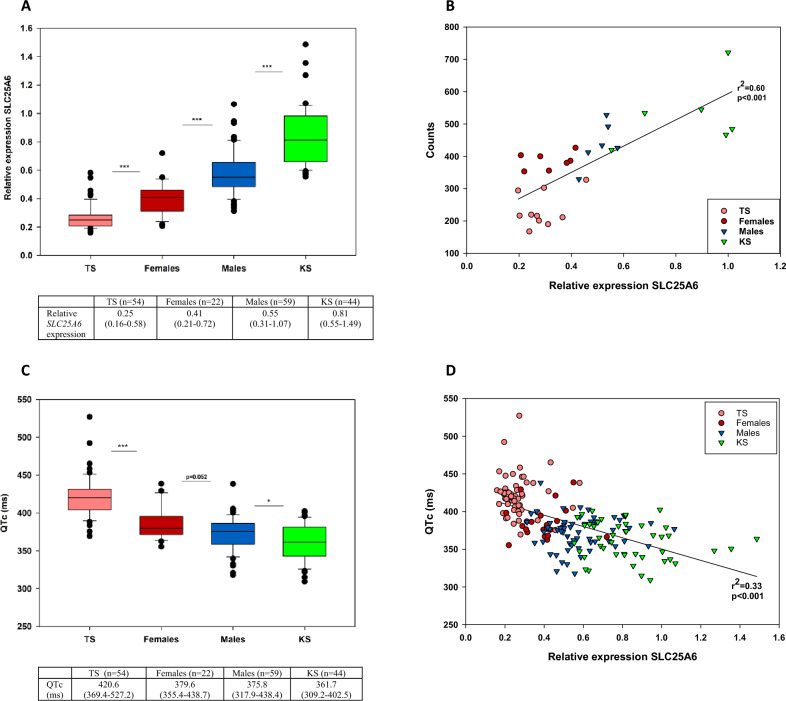
*SLC25A6* expression levels in humans correlate with QTc interval duration. (**A**) Box plot and table showing relative expression levels of *SLC25A6* determined by qPCR in patients with Turner syndrome, Klinefelter syndrome and female and male controls. Values in table are medians and range. n (TS) = 54, n(KS) = 44, n(females) = 22, n(males) = 59. (**B**) Correlations between absolute expression values of *SLC25A6* (counts) and relative expression of *SLC25A6* in the subpopulation. n(TS) = 10, n(KS) = 6, n(females) = 7, n(males) = 6). (**C**) Box plot and table of QTc in patients with Turner syndrome, Klinefelter syndrome and female and male controls. Values in the table are median and range. n(TS) = 54, n(KS) = 44, n(females) = 22, n(males) = 59. (**D**) Correlation between QTc and relative expression of *SLC25A6*. n(TS) = 54, n(KS) = 44, n(females) = 22, n(males) = 59). Corrected QT interval (QTc) was calculated using Bazett´s equation (QTc = QT × √1/RR). Statistical analysis were performed by Mann Whitney U-test (median with range).TS, Turner syndrome. KS, Klinefelter syndrome. *p < 0.05, ***p < 0.001.

To determine the effect of altered dosage of *SLC25A6* on QTc interval durations in humans, we first examined the relative expression level of *SLC25A6* in peripheral blood samples from patients with TS, KS and female and male controls using quantitative PCR (see Supplementary Fig. 2, Supplementary Table S1). In agreement with recent published studies^18,19^, we found that TS was associated with a significant downregulation of *SLC25A6*, whereas KS was associated with a significant upregulation of *SLC25A6* (Fig. 1A). We also found that *SLC25A6* was significantly upregulated in male controls compared to female controls (Fig. 1A). This is in agreement with a previous study demonstrating that *SLC25A6* exhibits a male-biased expression profile across human tissues, i.e., the expression profile mediated by an extension of the X inactivation process into the PAR region of the inactive X chromosome in individuals with more than one X chromosome^26^. To validate our data of relative expression levels, we assessed the expression level of *SLC25A6* using RNA sequencing in a subpopulation of patients with TS and KS and in female and male controls. A significant correlation between our relative expression levels and absolute expression levels of *SLC25A6* was seen (r^2^ = 0.60, p < 0.001) (Fig. 1B). To investigate if the expression pattern of *SLC25A6* seen in blood is representative of other human tissues of mesodermal origin (heart, aorta, muscle), we analyzed the expression level of *SLC25A6* using RNA sequencing in blood, muscle and aorta tissue from a subset of patients with TS and KS and female and male controls. The expression pattern of muscle tissue was comparable to that seen in blood with an upregulation in KS and downregulation in TS compared to controls (Supplementary Fig. 3) and analysis of the expression of *SCL25A6* in aorta from patients with TS showed an expression level equal to the expression level in blood seen in patients with TS (see Supplementary Fig. 3).

### *SLC25A6* expression levels in humans correlate with QTc interval duration

After having showed that *SLC25A6* expression levels correlate with sex-chromosome dosage in humans across tissue type of mesodermal origin, we next analyzed QTc interval duration from previously recorded ECGs in our cohort of patients with TS, KS and female and male controls to be able to correlate *SLC25A6* with QTc interval duration. We found that KS demonstrated the shortest QTc interval duration, followed by male controls and female controls, with TS showing the longest QTc interval duration (Fig. 1C). Correlation analysis of QTc interval duration and *SLC25A6* expression demonstrated a significant negative correlation (r^2^ =  − 0.33, p < 0.001) (Fig. 1D). However, the low r^2^ indicates that only a part of the variability seen in QTc interval can be explained by *SLC25A6* expression level.

### *SLC25A6* expression level impacts systolic duration and QTc interval duration in zebrafish

To gain further evidence of an impact of *SLC25A6* expression level on QTc interval, we confirmed cardiac slc25a6 expression by RT-PCR on a pool of isolated 3 days post fertilization (dpf) zebrafish hearts (see Supplementary Fig. 4) and recorded the effect of *SLC25A6* overexpression and *slc25a6* downregulation on systolic contraction duration in 3 days post fertilization (dpf) zebrafish larvae using high-speed video recordings. The physiological condition of KS carrying three copies of *SLC25A6* was modeled in zebrafish embryos by overexpression of wildtype *SLC25A6* by mRNA microinjection into newly fertilized zebrafish embryos. RT-PCR analysis indicated similar in vivo stability of human wildtype *SLC25A6* mRNA and full length control SLC25A6 mRNA containing two premature stop codons (see Supplementary Fig. 5). The physiological condition of TS carrying only one copy of *SLC25A6* was modeled in zebrafish embryos by *slc25a6* knockdown titrated to approximately 50% downregulation of the wildtype endogenous *slc25a6* mRNA level (see Supplementary Fig. 6). Neither *SLC25A6* overexpression nor *slc25a6* downregulation overtly affected zebrafish embryo morphology (see Supplementary Fig. 7).

**Figure 2:**
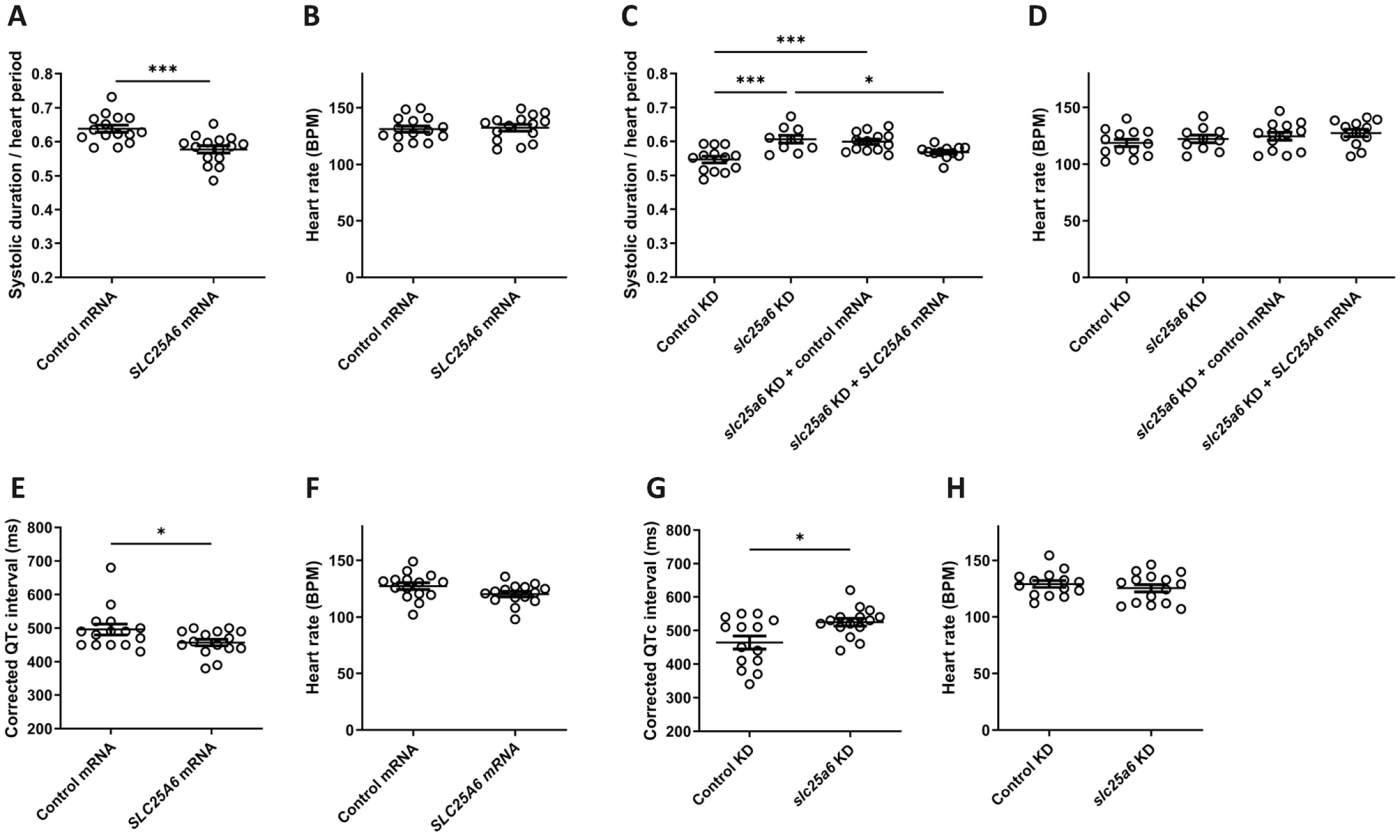
*SLC25A6* expression level impacts systolic duration and QTc interval duration in zebrafish. (**A**) Systolic interval normalized to heart period determined from high-speed video recordings of the heart ventricle show reduced systolic duration resulting from *SLC25A6* overexpression (SLC25A6 mRNA) compared to controls (Control mRNA). (**B**) *SLC25A6* overexpression did not affect heart rate. Data in (**A,B**) were compiled from three independent experiments, each encompassing both groups (n = 15). (**C**) Systolic interval normalized to heart period determined from high-speed video recordings of the heart ventricle show increased systolic duration resulting from approximately 50% knockdown of endogenous *slc25a6* mRNA (*slc25a6* KD) compared to controls (Control KD). The phenotype was rescued by co-injection of the *slc25a6* targeted morpholino with wildtype *SLC25A6* mRNA (*slc25a6* KD + *SLC25A6* mRNA) but not control mRNA (*slc25a6* KD + control mRNA). (**D**) Knockdown of endogenous *slc25a6* mRNA did not affect heart rate. Data in (**C,D**) were compiled from three independent experiments, each encompassing all four groups. n(control KD) = 13, n(*slc25a6* KD) = 10, n(*slc25a6* KD + control mRNA) = 13, n(*slc25a6* KD + *SLC25A6* mRNA) = 12. (**E**) *SLC25A6* overexpression (*SLC25A6* mRNA) resulted in a decreased QTc interval compared to controls (Control mRNA). (**G**) 50% downregulation of endogenous *slc25a6* mRNA (*slc25a6* KD) resulted in an increased QTc interval compared to controls (Control KD). (**F**,**H**) S*LC25A6* overexpression (*SLC25A6* mRNA) and downregulation of endogenous *slc25a6* (*slc25a6* KD) did not affect heart rate. n = 15 (Control mRNA), n = 15 (*SLC25A6* mRNA), n = 14 (Control KD), n = 1 (*slc25a6* KD). Corrected QT interval (QTc) was calculated using Bazett’s equation (QTc = QT × √1/RR). Statistical analyses were performed by one-way ANOVA with Tukey’s post-test. *p < 0.05, ***p < 0.001.

We observed that in zebrafish with overexpression of *SLC25A6*, a significant shortening of systolic duration occurred compared to controls (Fig. 2A). In addition, *slc25a6* downregulation resulted in a significant prolongation of systolic duration compared to controls (Fig. 2C). No effect on heart rate was seen (Fig. 2B,D,F,H). The specificity of the phenotype of *slc25a6* downregulated embryos was substantiated by phenotypic rescue resulting from the co-injection of wildtype *SLC25A6* mRNA, but not control mRNA (Fig. 2C). Importantly, ECG analysis of 3 days post fertilization zebrafish larvae revealed a significantly shortened and prolonged QTc interval duration by *SLC25A6* overexpression and *slc25a6* downregulation, respectively (Fig. 2E,G), demonstrating that the observed changes in systolic duration correspond to altered QTc interval duration. The averaged ECG recordings and representative ECG traces are shown in Supplementary Fig. 9. Collectively, these findings show that that the level of *SLC25A6* expression impact QTc interval duration in vivo, thereby adding futher evidence of an impact of *SLC25A6* expression level on QTc interval.

### Pharmacological rescue of *SLC25A6* overexpression and *slc25a6* knockdown phenotypes

**Figure 3:**
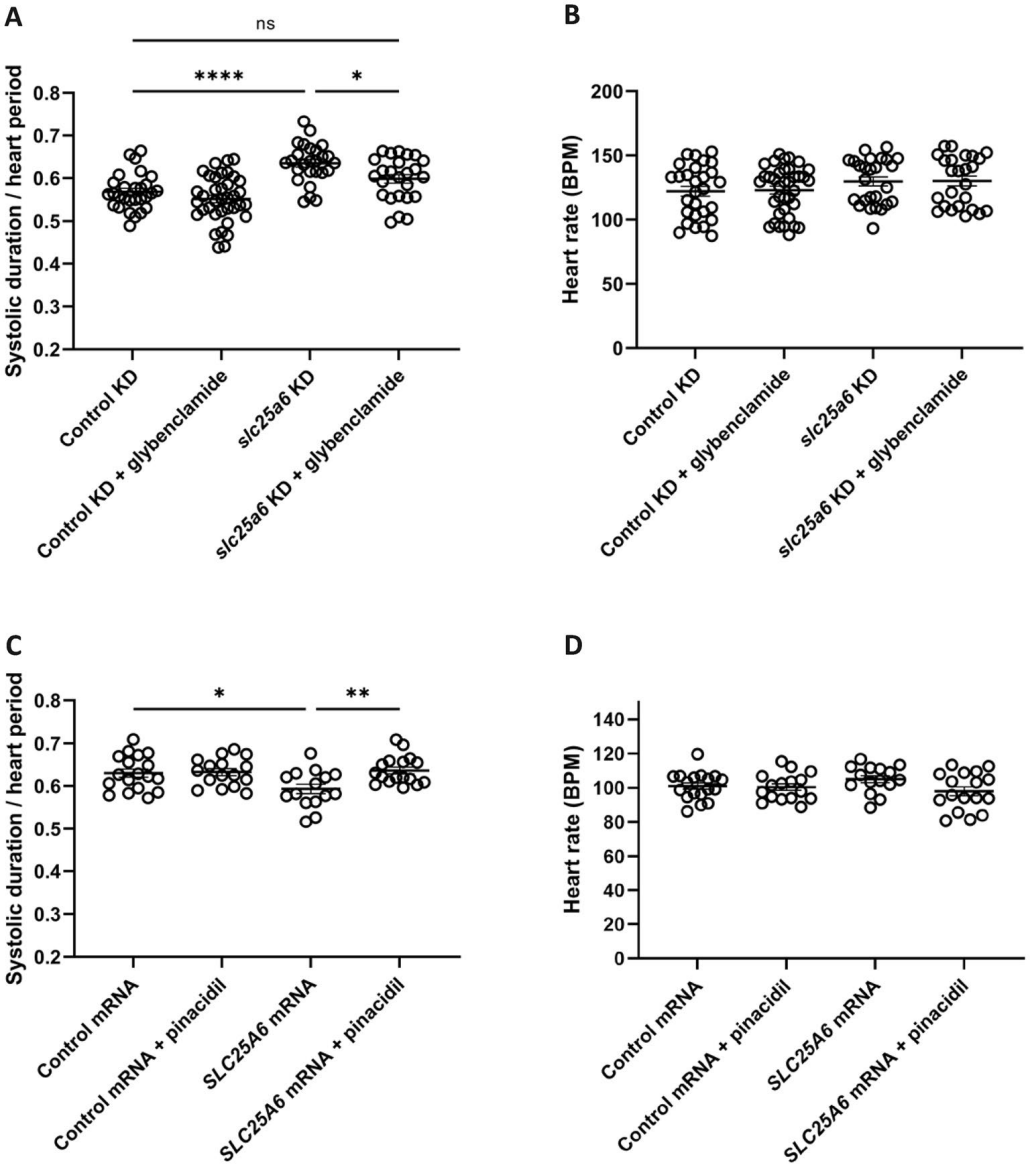
Pharmacological rescue of *SLC25A6* overexpression and *slc25a6* knockdown phenotypes. (**A**) Systolic duration normalized to heart period determined from high-speed video recordings of the heart ventricle. Systolic interval prolongation induced by *slc25a6* knockdown (*slc25a6* KD) is normalized by glybenclamide treatment (*slc25a6* KD + glybenclamide). (**B**) Neither *slc25a6* knockdown (slc25a6 KD) nor glybenclamide treatment (control KD + glybenclamide and *slc25a6* KD + glybenclamide) affects heart rate. Data in (**A,B**) were compiled from four independent experiments, each encompassing all four groups. n(Control KD) = 28, n(Control KD + glybenclamide) = 37, n(*slc25a6* KD) = 26, n(*slc25a6* KD + glybenclamide) = 25. (**C**) Systolic interval normalized to heart period determined from high-speed video recordings of the heart ventricle. Systolic interval shortening induced by *SLC25A6* overexpression (*SLC25A6* mRNA) is diminished by pinacidil treatment (*SLC25A6* mRNA + pinacidil). (**D**) Neither *SLC25A6* overexpression (*SLC25a6* mRNA) nor pinacidil treatment (Control mRNA + pinacidil and *SLC25A6* mRNA + pinacidil) affects heart rate. Data in (**C,D**) were compiled from three independent experiments, each encompassing all four groups. n(Control mRNA) = 18, n(Control mRNA + pinacidil) = 16, n(*SLC25A6* mRNA) = 15, n(*SLC25A6* mRNA + pinacidil) = 17. Statistical analyses were performed by one-way ANOVA with Tukey´s post-test. *p < 0.05, **p < 0.01, ****p < 0.0001.

As *SLC25A*6 doses can modify intracellular ADP/ATP ratio through its ADP/ATP antiporter activity, we hypothesized an involvement of the K_ATP_ channel. To test this hypothesis, we treated *slc25a6* knockdown zebrafish with the K_ATP_ channel antagonist glybenclamide and quantified systolic duration. We observed that glybenclamide treatment resulted in a normalization of the systolic duration (Fig. 3A). Heart rate was unaffected by glybenclamide treatment (Fig. 3B) and no pharmacological effect of glybenclamide on controls was observed (p = 0.50) (Fig. 3A), in accordance with the majority of K_ATP_ channels consitutevely closed under resting conditions^27^. Treatment with K_ATP_ channel agonist pinacidil normalized the shortened systolic interval duration induced by *SLC25A6* overexpression with no pharmacological effect on controls (Fig. 3C). Heart rate was unaffected by pinacidil treatment (Fig. 3D). Collectively, these results show, that prolonged or shortened systolic interval caused by altered *SLC25A6* expression can be modulated pharmacologically.

## Discussion

This study finds evidence of the first pseudoautosomal gene, *SLC25A6*, contributing to QTc interval duration. We showed a significant negative correlation between the expression of *SLC25A6* and QTc interval in humans. Our zebrafish model supported these findings and further revealed that pharmacological intervention was possible. Our data not only provide evidence that *SLC25A6* contributes to QT interval variation in the general population, but also that *SLC25A6* is involved in the cardiac phenotypes with altered QTc interval seen in TS and KS.

The significant correlation found between the expression level of *SLC25A6* and QTc interval across humans adds *SLC25A6* to the list of genes contributing to the variation in QT interval seen in the human population. Previously, genome-wide association (GWAS) studies have contributed to the identification of common autosomal variants, which explain up to 8–10% of QT interval variation in the general population. However, one of the limitations of previously performed GWAS studies is the inclusion of only autosomal variants, an approach overlooking the possibility that pseudoautosomal genes and X chromosomal genes could contribute to QT interval variation. Our study highlights the role of the pseudoautosomal gene *SLC25A6* in explaining QT interval variation; however other pseudoautosomal and X chromosomal genes may also be involved.

The rescuing effect of the K_ATP_ channel agonist pinacidil and the K_ATP_ channel antagonist glybenclamide on systolic interval duration in our zebrafish model indicated that expression levels of *SLC25A6* might indirectly affect the function of the K_ATP_ channel in the cardiomyocytes through alterations of ATP/ADP levels. The role of K_ATP_ channels in cardiac electrical activity is rather complex and depends on their localization^28,29^. The K_ATP_ channels in ventricle cardiomyocytes are generally considered cardioprotective metabolic sensors that activate upon acute ischemic conditions and shorten the action potential duration and thus QT interval. These changes normally occur because of severe changes in intracellular ATP upon metabolic impairment. In contrast, the K_ATP_ channels of the cardiac conduction system have a different molecular composition that makes them more readily activated by ADP and less sensitive to inhibitory action of ATP, i.e., sensitive to minor changes in ADP and ATP^30^. Activation of these K_ATP_ channels may slow the conduction velocity and thus, prolong the systolic duration and QT interval^31^ as seen under chronic ischemic conditions^32^. Moreover, gain-of-function mutations in the K_ATP_ channel were suggested to prolong repolarization in some (epicardial) cardiomyocytes^33^, as well as increasing potassium transmembrane gradient, while reduction of the gradient shortened action potential^34^.

In recent years, our knowledge of the biology of ADP/ATP translocases has expanded. In addition to their involvement in ATP/ADP exchange, evidence also suggests that ADP/ATP translocases are targets of mitochondrial uncouplers^35^ and involved in mitochondrial H^+^ leak^36^, mitochondrial membrane potential maintenance^37^, and potentiation of the mitochondrial permeability transition pore^38^. It is therefore possible, that the alterations seen in QTc interval with altered *SLC25A6* expression, are due to a more general impact of *SLC25A6* on the mitochondrial function, and not an isolated effect on the K_ATP_ channel. Future studies are needed to demonstrate the mechanistic contribution of the ADP/ATP translocase to electrical function of cardiomyocytes.

Further studies will be needed to decipher the molecular mechanisms and physiological basis of the *SLC25A6* dosage and expression level-dependent regulation of QT interval variation.

In conclusion, our study highlights that the dosage and expression level of the pseudoautosomal gene *SLC25A6* contribute to QTc interval variation.

## Methods

### Human subjects

The human subjects were recruited as previously described^11,13^. Males with verified KS (n = 44) were recruited through endocrine and clinical genetic hospital outpatient clinics as well as fertility clinics in Denmark, whereas women with verified TS (n = 54) were recruited through the Danish National Society of Tuner Syndrome Contact Group and an endocrine outpatient clinic in Denmark. Healthy, age-matched men (n = 59) and women (n = 22) were recruited through advertisement to serve as controls. The subpopulation included males with verified KS (n = 6), women with verified TS (n = 10), healthy, age-matched men (n = 6) and women (n = 7). The validation cohort included males with verified KS (n = 16), women with verified TS (n = 10), healthy men (n = 16) and women (n = 5), all included in the main population. Participants were recruited as described above.

### Relative quantification of *SLC25A6* expression in peripheral blood from human subjects

Two and a half ml of peripheral blood were collected in PaxGene blood RNA tubes (total volume 10 ml) (BD, Franklin Lakes, NJ, USA) followed by extraction of total RNA using PaxGene Blood RNA Kit (Qiagen, Hilden, Germany) according to the manufacturer’s protocol including DNAase I treatment to remove residual genomic DNA. The RNA was eluted in 40 µl of nuclease-free water, quantified using Dropsense96 (PerkinElmer, Waltham, MA, USA) and stored at − 80 °C. Subsequently, 500 ng of RNA was converted to cDNA using SuperScript II reverse transcriptase (ThermoFisher, Waltham, MA, USA) according to the manufacturers recommendations and a combination of Oligo(dT)15 (250 ng) (5ʹ-TTT TTT TTT TTT TTT VN-3ʹ, V = A, G or C, and N = A, T, G or C (SigmaAldrich, St Louis, MO, USA)) and random hexamer primers (Invitrogen, Carlsbad, CA, USA) (125 ng; reaction volume 20 µl). The expression of *SLC25A6* and *Ubiquitin* C (*UBC*) (endogenous reference gene) were measured using quantitative PCR (qPCR). The qPCR reactions were performed in triplicates on a ViiA 7 Real-Time PCR instrument (Applied Biosystems, Thermofisher, Waltham, MA, USA) according to the manufacturer’s instructions and using the following TaqMan gene expression assays (ThermoFisher): Hs00745067_s1 (*SLC25A6*) and Hs00824723_m1 (*UBC*). 2 μl of each cDNA (diluted 50-fold) was used as a template in the qPCR reactions together with 8 μl Gene Expression Master Mix (reaction volume 10 µl). Representative qPCR curves are illustrated in Supplementary Fig. 2). Raw C_T_ values (Supplementary Table S1) were calculated with automatic baseline and threshold settings, and the relative expression levels of *SLC25A6* were determined using the comparative C_T_ method (2^−ΔΔCT^ method), i.e., normalization to the reference gene (*UBC*) and relative to an internal calibrator sample using the QuantStudio Real-Time PCR Software v1.3 (Thermofisher).

### Whole blood *SLC25A6* expression in the subpopulation

*SLC25A6* expression was assessed in a separate cohort based on RNA sequencing of whole blood from females with verified TS and healthy women (Trolle et al.^39^, data accessible at the European Genome-Phenome Archive (EGA), EGAS00001002190), and males with verified KS and healthy men (Skakkebæk et al.^21^, data accessible at the EGA, EGAS00001002797). Paired de-multiplexed fastq files were subjected to initial quality control using FastQC (Babraham Bioinformatics). The adaptor removal, in addition to trimming of low-quality ends, was then conducted using Trim Galore with default settings (Babraham Bioinformatics), followed by mapping to the human genome (hg38) using HISAT2^40^. The overlaps between genomic annotations (Gencode v 37) and the read alignments were quantified by feature Counts to produce gene counts for each sample^41^. The read counts were then normalized by the R Bioconductor package DESeq2^42^, and the estimated gene expression of *SLC25A6* for each sample was used for correlation analysis.

### Whole blood and muscle tissue expression of *SLC25A6* in the validation cohort

SLC25A6 expression was assessed in a separate cohort based on RNA sequencing of whole blood and muscle from females with verified TS, males with verified KS and healthy women and men^43^. The RNA-seq libraries were multiplexed paired-end sequenced on a Illumina NovaSeq 6000 (100 bp). Paired de-multiplexed fastq files were subjected to initial quality control using FastQC (Babraham Bioinformatics). Adaptor removal and trimming of low-quality ends were then conducted using Trim Galore with default settings (Babraham Bioinformatics). Gene expression was quantified by quasi-mapping using Salmon^44^. A decoy-aware transcriptome index was built based on the hg38 transcriptome and selective alignment was run using the fastq pairs as input. Transcript abundancies were summarized to gene-level using the R package Tximeta^45^. The gene counts were then normalized by the R Bioconductor package DESeq2^42^, and the estimated gene expression of *SLC25A6* for each sample was used for correlation analysis.

### Aortic expression of *SLC25A6*

Aortic biopsies were collected from three patients with TS undergoing planned elective thoracic surgery due to aortic dilatation. The biopsies were immediately cleaned and snap-frozen in liquid nitrogen and stored at − 80 °C. The frozen biopsies were dissociated using a CovarisSP02 and RNA was extracted by the AllPrep DNA/RNA/PROTEIN Mini kit 8004 (Qiagen). Synthesis of directional RNA-seq libraries were conducted as described above using the KAPA RNA HyperPrep with RiboErase kit (HMR) (Roche).

### Electrocardiogram and QTc measurements in human subjects

Twelve lead electrocardiograms (ECG) from females with verified TS and healthy women, and, males with verified KS and healthy men were obtained as described previously^11,13^. ECGs were recorded at 25 mm/s with an amplitude of 10 mm/mV (Personal 120/210 machine (Esaote Biomedica, Cambridge, United Kingdom) and scanned to a digital file. Cardio Calipers 3.3. (Iconico, http://www.iconico.com) were used to measure QT interval and RR interval. The corrected QT interval (QTc) was calculated using Bazett´s equation (QTc = QT × √1/RR)^6^.

### Zebrafish

Adult AB wildtype zebrafish originating from the European Zebrafish Resource Center (Karlsruhe, Germany) were maintained on commercial recirculating zebrafish housing systems (Tecniplast, Buguggiate, Italy) in reverse osmosis water conditioned to 0.7mS, pH 7.2, 28 °C, on a 14 h light/10 h darkness cycle and fed four times daily. Embryos were obtained by natural crosses and reared in E3 medium (5 mM NaCl, 0.17 mM KCl, 0.33 mM CaCl_2_, 0.33 mM MgSO_4_, 10^–5^% methylene blue, 2 mM HEPES adjusted to pH 7.4 with NaHCO_3_) at 28 °C.

### Overexpression of *SLC25A6* and knockdown of *slc25a6* in zebrafish

To evaluate embryonic cardiac *slc25a6* expression, hearts from 12.3 days post fertilization (dpf) zebrafish embryos were isolated by manual microdissection and pooled in RNAlater (Sigma). RNA was isolated using Qiagen RNeasy Plus Micro Kit. RT-PCR was performed using Qiagen Onestep RT-PCR Kit and primers pairs 5ʹ-CACGAGACCACCTTCAACT-3ʹ/5ʹ-ATCCAGACGGAGTATTTGC-3ʹ (*β-actin*) and 5ʹ-TGCAGCCTTTTCTTTTGCGA-3ʹ/5ʹ-AATGGGTAGGAGACCACGCC-3ʹ (*slc25a6*) followed by a nested PCR reaction using primer pair 5ʹ-CAGATGGCTTACGAGGGCTG-3ʹ/5ʹ-TGTGGGTGTTCTTTGGATCGG-3ʹ (*slc25a6*). PCR product identity were verified by Sanger sequencing. Primer pairs 5ʹ AATTAATACGACTCACTATAGAACATGACGGAACAGGCCATCTC/CACACTGGACTAGTGGATCCTTAG-3ʹ and 5ʹ AATTAATACGACTCACTATAGAACATGACGTAATAGGCCATCTC-3ʹ/5ʹ-CACACTGGACTAGTGGATCCTTAG-3ʹ were used to generate T7-promoter-containing Kozak-optimized templates for the synthesis of mRNA encoding wildtype human *SLC25A6* and a full length control *SLC25A6* transcript containing two premature stop codons (SLC25A6 E3stop,Q4stop), using Genscript clone OHu19558 as a template. mRNAs were synthesized using mMessage mMachine T7 ULTRA kit (Ambion) according to suppliers’ recommendations and microinjected at 400 pg per embryo into freshly fertilized zebrafish embryos no later than the two-cell stage. The stability of injected transcripts 3 days post fertilization was assessed by RT PCR using primers: 5ʹ-CAAGAACACGCACATCGTGG-3ʹ/5ʹ-GTACAGGACCAGCACGAAGG-3ʹ and *β-actin* mRNA levels were assessed using the primer pair 5ʹ-CACGAGACCACCTTCAACT-3ʹ/5ʹ-ATCCAGACGGAGTATTTGC-3ʹ. PCR product identities were verified by Sanger sequencing. *slc25a6* knockdown was performed by microinjection of *slc25a6* exon2-intron2 targeted morpholino (TCACAAACCTATTACCTTTAGCAGT) into newly fertilized zebrafish embryos no later than the two-cell stage. The morpholino was titrated to obtain an approximately 50% reduction of endogenous *slc25a6* mRNA levels at 3 dpf. Standard control morpholino (CCTCTTACCTCAGTTACAATTTATA, Gene Tools, LLC) was injected in equimolar amount as a negative control. Morpholino efficiency was assessed by reverse transcription PCR (RT-PCR) from cDNA synthesized from total RNA extracted from groups of 10 microinjected zebrafish larvae three days post fertilization using the primer pair 5ʹ-GCGCCGATTGAGAGAGTCAA-3ʹ/5ʹ-AATGGGTAGGAGACCACGCC-3ʹ and PCR product identities were verified by Sanger sequencing. Band intensities were determined using ImageJ^46^.

### Imaging and measurements of cardiac function and ECG in zebrafish

Whole-mount images were done on 3 days post fertilization zebrafish larvae anaesthetized with 150 ng/ml ethyl 3-aminobenzoate methanesulfonate (150 ng/ml; MS-222; Sigma-Aldrich) in E3 medium and staged in 3% hydroxypropyl methylcellulose (average Mn ~ 86,000, Sigma Aldrich) in E3 medium. High-speed video recordings of cardiac function of zebrafish larvae were performed (Supplementary Fig. 8) and analyzed as previously described^47^.

ECG recordings in 3 days post fertilization zebrafish larvae were performed as described previously^47^. Zebrafish larvae were anaesthetized using 150 ng/ml MS-222 (Sigma) in E3 medium at room temperature (22–24 °C). A borosilicate glass micropipette (PG15OT-7.5; Harvard Apparatus) filled with 3 mM KCl with tip resistances of approximately 10 MΩ was positioned on larvae skin surface (no penetration). Data acquisition was done at 10 kHz using an Axopatch 200B amplifier (Axon Instruments, Inc.) in a current-clamp configuration with the software package Clampex 7 (Axon Instruments Inc.). Data analysis was automated in ECG analysis module of LabChart 8 software (ADInstruments) (Supplementary Fig. 9). QT interval and RR interval were automatically calculated for each larva and the corrected QT interval (QTc) was assessed using Bazett’s equation (QTc = QT/√RR, where RR is the interval between two consequent R waves in ECG)^6^.

### Pharmacological interventions with glybenclamide and pinacidil in zebrafish

For pharmacological rescue experiments, 3 days post fertilization zebrafish larvae were treated with 50 µM pinacidil (Sigma-Aldrich P154), 50 µM glybenclamide (Sigma-Aldrich G0639) in 0.1% DMSO or vehicle control combined with 150 ng/ml MS-222 (Sigma) anesthesia in E3 medium 15–30 min prior to high-speed recordings.

### Statistics

Statistical analyses were conducted using SPSS 21.0 (IBM Corp., Armonk, NY, USA) and GraphPad Prism 7 (GraphPad Software, San Diego, CA). Comparisons of continuous variables were performed using Student’s independent t test (mean ± SD) or one-way ANOVA with Tukey´s post-test for normally distributed variables, and Mann Whitney U-test (median with range) was used for non-parametric variables.

### Study approval

The study was approved by The Danish Data Protection Agency and the local and central ethics committee (Region Midtjylland, Denmark number M-20080238 and M-20010248, Central Denmark Regional Committee on Health Research Ethics number 1-10-72-131-15) and registered at ClinicalTrials.gov (NCT00624949, NCT00999310, NCT02526628). All participants provided informed consent. All methods were performed in accordance with the relevant guidelines and regulations. All experimental zebrafish procedures were performed in agreement with Danish legislation and guidelines from the European Convention for the Protection of Vertebrate Animals used for Experimental and other Scientific Purposes. The study was reported in accordance with ARRIVE guidelines.

## Supplementary Information

Supplementary Table S1.

Supplementary Figures.

## Data Availability

The data supporting the findings of this study are available from the corresponding author upon reasonable request. The RNA sequencing datasets used in this study are available at the European Genome-Phenome Archive (EGA) (EGAS00001002190, EGAS00001002797, EGAS00001006404).

## References

[1] Scrocco C, Bezzina CR, Ackerman MJ, Behr ER (2021). Genetics and genomics of arrhythmic risk: Current and future strategies to prevent sudden cardiac death. Nat. Rev. Cardiol..

[2] Giudicessi JR, Ackerman MJ (2012). Potassium-channel mutations and cardiac arrhythmias-diagnosis and therapy. Nat. Rev. Cardiol..

[3] Arking DE (2014). Genetic association study of QT interval highlights role for calcium signaling pathways in myocardial repolarization. Nat. Genet..

[4] Newton-Cheh C (2009). Common variants at ten loci influence QT interval duration in the QTGEN study. Nat. Genet..

[5] Noseworthy PA (2011). Common genetic variants, QT interval, and sudden cardiac death in a Finnish population-based study. Circ. Cardiovasc. Genet..

[6] Bazett HC (1920). An analysis of the time-relations of electrocardiograms. Heart.

[7] Offerhaus JA, Bezzina CR, Wilde AAM (2020). Epidemiology of inherited arrhythmias. Nat. Rev. Cardiol..

[8] Gershoni M, Pietrokovski S (2017). The landscape of sex-differential transcriptome and its consequent selection in human adults. BMC Biol..

[9] Oliva M (2020). The impact of sex on gene expression across human tissues. Science.

[10] Bondy CA (2006). Prolongation of the cardiac QTc interval in Turner syndrome. Medicine.

[11] Trolle C (2013). Long QT interval in Turner syndrome—A high prevalence of LQTS gene mutations. PLoS ONE.

[12] Sozen AB (2008). Atrial and ventricular arryhthmogenic potential in Turner syndrome. Pace.

[13] Jorgensen IN (2015). Short QTc interval in males with Klinefelter syndrome-influence of CAG repeat length, body composition, and testosterone replacement therapy. Pacing Clin. Electrophysiol..

[14] Zitzmann M (2015). Gene expression patterns in relation to the clinical phenotype in Klinefelter syndrome. J Clin. Endocrinol. Metab..

[15] Slim R (1993). A human pseudoautosomal gene encodes the ANT3 ADP/ATP translocase and escapes X-inactivation. Genomics.

[16] Stepien G, Torroni A, Chung AB, Hodge JA, Wallace DC (1992). Differential expression of adenine nucleotide translocator isoforms in mammalian tissues and during muscle cell differentiation. J. Biol. Chem..

[17] Karlsson M (2021). A single-cell type transcriptomics map of human tissues. Sci. Adv..

[18] Zhang X (2020). Integrated functional genomic analyses of Klinefelter and Turner syndromes reveal global network effects of altered X chromosome dosage. Proc. Natl. Acad. Sci. U.S.A..

[19] Manotas MC (2020). Identification of common differentially expressed genes in Turner (45, X) and Klinefelter (47, XXY) syndromes using bioinformatics analysis. Mol. Genet. Genom. Med..

[20] Belling K (2017). Klinefelter syndrome comorbidities linked to increased X chromosome gene dosage and altered protein interactome activity. Hum. Mol. Genet..

[21] Skakkebaek A (2018). DNA hypermethylation and differential gene expression associated with Klinefelter syndrome. Sci. Rep..

[22] Howe K (2013). The zebrafish reference genome sequence and its relationship to the human genome. Nature.

[23] Nemtsas P, Wettwer E, Christ T, Weidinger G, Ravens U (2010). Adult zebrafish heart as a model for human heart? An electrophysiological study. J. Mol. Cell. Cardiol..

[24] Echeazarra L, Hortigon-Vinagre MP, Casis O, Gallego M (2020). Adult and developing zebrafish as suitable models for cardiac electrophysiology and pathology in research and industry. Front. Physiol..

[25] Vornanen M, Hassinen M (2016). Zebrafish heart as a model for human cardiac electrophysiology. Channels.

[26] Tukiainen T (2017). Landscape of X chromosome inactivation across human tissues. Nature.

[27] Kantor PF, Coetzee WA, Carmeliet EE, Dennis SC, Opie LH (1990). Reduction of ischemic K+ loss and arrhythmias in rat hearts. Effect of glibenclamide, a sulfonylurea. Circ. Res..

[28] Nichols CG (2016). Adenosine triphosphate-sensitive potassium currents in heart disease and cardioprotection. Card. Electrophysiol. Clin..

[29] Foster MN, Coetzee WA (2016). KATP Channels in the cardiovascular system. Physiol. Rev..

[30] Bao L (2011). Unique properties of the ATP-sensitive K(+) channel in the mouse ventricular cardiac conduction system. Circ. Arrhythm. Electrophysiol..

[31] Tse G, Chan YW, Keung W, Yan BP (2017). Electrophysiological mechanisms of long and short QT syndromes. Int. J. Cardiol. Heart Vasc..

[32] Beinart R (2014). The QT interval is associated with incident cardiovascular events: The MESA study. J. Am. Coll. Cardiol..

[33] Kusano KF (2013). Brugada syndrome: Recent understanding of pathophysiological mechanism and treatment. J. Arrhythmia.

[34] Yan GX, Antzelevitch C (1998). Cellular basis for the normal T wave and the electrocardiographic manifestations of the long-QT syndrome. Circulation.

[35] Bertholet AM (2022). Mitochondrial uncouplers induce proton leak by activating AAC and UCP1. Nature.

[36] Bertholet AM (2019). H(+) transport is an integral function of the mitochondrial ADP/ATP carrier. Nature.

[37] Ouyang Y (2022). Phosphate starvation signaling increases mitochondrial membrane potential through respiration-independent mechanisms. BioRxiv.

[38] Karch J (2019). Inhibition of mitochondrial permeability transition by deletion of the ANT family and CypD. Sci. Adv..

[39] Trolle C (2016). Widespread DNA hypomethylation and differential gene expression in Turner syndrome. Sci. Rep..

[40] Kim D, Langmead B, Salzberg SL (2015). HISAT: A fast spliced aligner with low memory requirements. Nat. Methods.

[41] Liao Y, Smyth GK, Shi W (2014). featureCounts: An efficient general purpose program for assigning sequence reads to genomic features. Bioinformatics.

[42] Love MI, Huber W, Anders S (2014). Moderated estimation of fold change and dispersion for RNA-seq data with DESeq2. Genome Biol..

[43] Johannsen EB (2022). Sex chromosome aneuploidies give rise to changes in the circular RNA profile: A circular transcriptome-wide study of Turner and Klinefelter syndrome across different tissues. Front. Genet..

[44] Patro R, Duggal G, Love MI, Irizarry RA, Kingsford C (2017). Salmon provides fast and bias-aware quantification of transcript expression. Nat. Methods.

[45] Love MI (2020). Tximeta: Reference sequence checksums for provenance identification in RNA-seq. PLoS Comput. Biol..

[46] Schneider CA, Rasband WS, Eliceiri KW (2012). NIH Image to ImageJ: 25 years of image analysis. Nat. Methods.

[47] Thorsen K (2017). Loss-of-activity-mutation in the cardiac chloride-bicarbonate exchanger AE3 causes short QT syndrome. Nat. Commun..

