# Supplementary material for: Dosage of the pseudoautosomal gene SLC25A6 is implicated in QTc interval duration
Source: Sci Rep. 2023 Jul 26;13:12089. doi: 10.1038/s41598-023-38867-3 (PMC10372092; doi:10.1038/s41598-023-38867-3)
Supplement: Supplementary file 2 — Supplementary Figures. [file 41598_2023_38867_MOESM2_ESM.pdf]

Supplementary Information

Dosage of the pseudoautosomal gene *SLC25A6* is implicated in QTc interval duration

Anne Skakkebæk[1,2,3], Kasper Kjær-Sørensen[4], Vladimir V. Matchkov[5], Lise-Lotte Christensen[2,3], Jesper Just[2,3], Cagla Cömert[6], Niels Holmark Andersen[7], Claus Oxvig[4], Claus Højbjerg Gravholt[2,3,8]

[1]Department of Clinical Genetics, Aarhus University Hospital, Aarhus, Denmark

[2]Department of Molecular Medicine, Aarhus University Hospital, Aarhus, Denmark

[3]Department of Clinical Medicine, Aarhus University Hospital, Aarhus, Denmark

[4]Department of Molecular Biology and Genetics, Aarhus University, Aarhus, Denmark

[5]Department of Biomedicine, Aarhus University, Aarhus, Denmark

[6]Research Unit for Molecular Medicine, Department of Clinical Medicine, Aarhus University and Aarhus University Hospital, Aarhus, Denmark

[7]Department of Cardiology, Aalborg University Hospital, Aalborg, Denmark

[8]Department of Endocrinology and Internal Medicine and Medical Research Laboratories, Aarhus University Hospital, Aarhus, Denmark

Corresponding author:

Anne Skakkebæk, Department of Clinical Genetics and Department of Molecular Medicine and Department of Clinical Medicine, Aarhus University Hospital, Palle Juul-Jensens Boulevard 99, 8200 Aarhus N, Denmark, telephone number: +45 27212998, email: asj@clin.au.dk

A

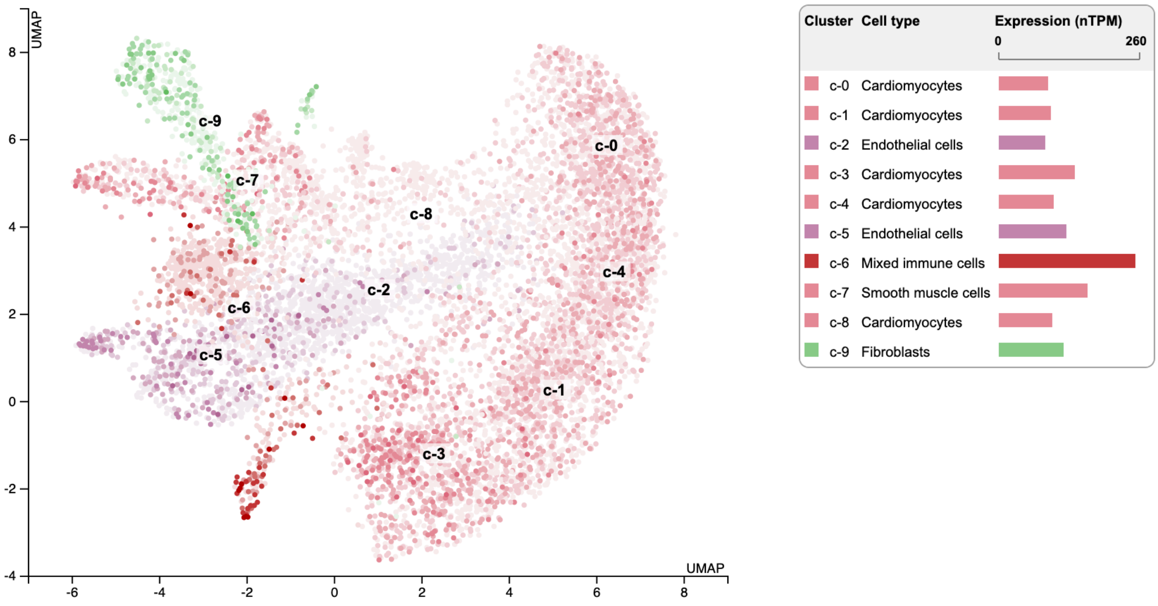

B

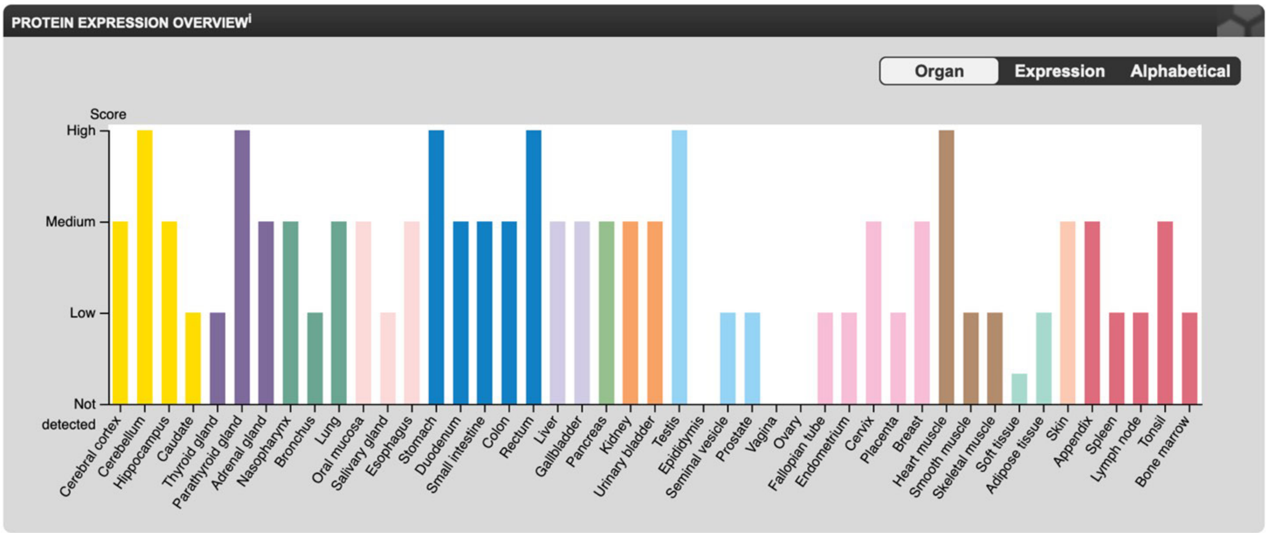

Supplemental Figure 1. (A) UMAP clustering of the different cell types in human heart tissue and the corresponding expression of *SLC25A6* within each cell type (proteinatlas.org). This plot was based on the integration and analysis of publicly available single cell RNAseq (scRNA-seq) datasets (https://www.proteinatlas.org/ENSG00000169100-SLC25A6/single+cell+type/heart+muscle). (B) Protein abundance of *SLC25A6* in all available tissue (proteinatlas.org). *SLC25A6* protein has been detected in the majority of the tissues tested, albeit with different intensity. A high protein abundance was observed in heart muscle tissue.

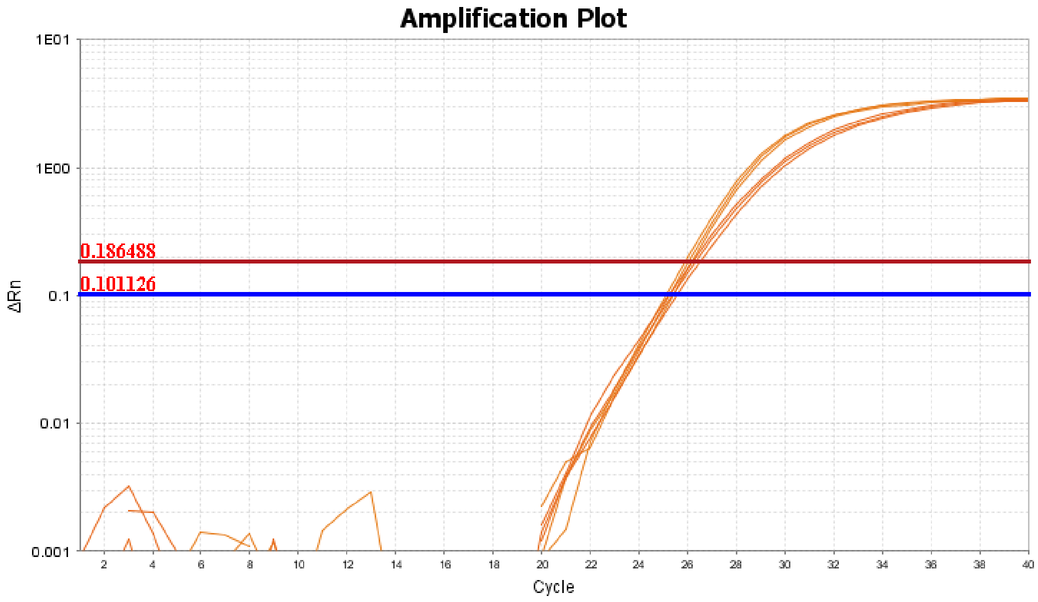

Supplemental Figure 2. Representative qPCR curves for *SLC25A6* and *UBC*. Red line, threshold for *SCL25A6*. Blue line, threshold for *UBC*.

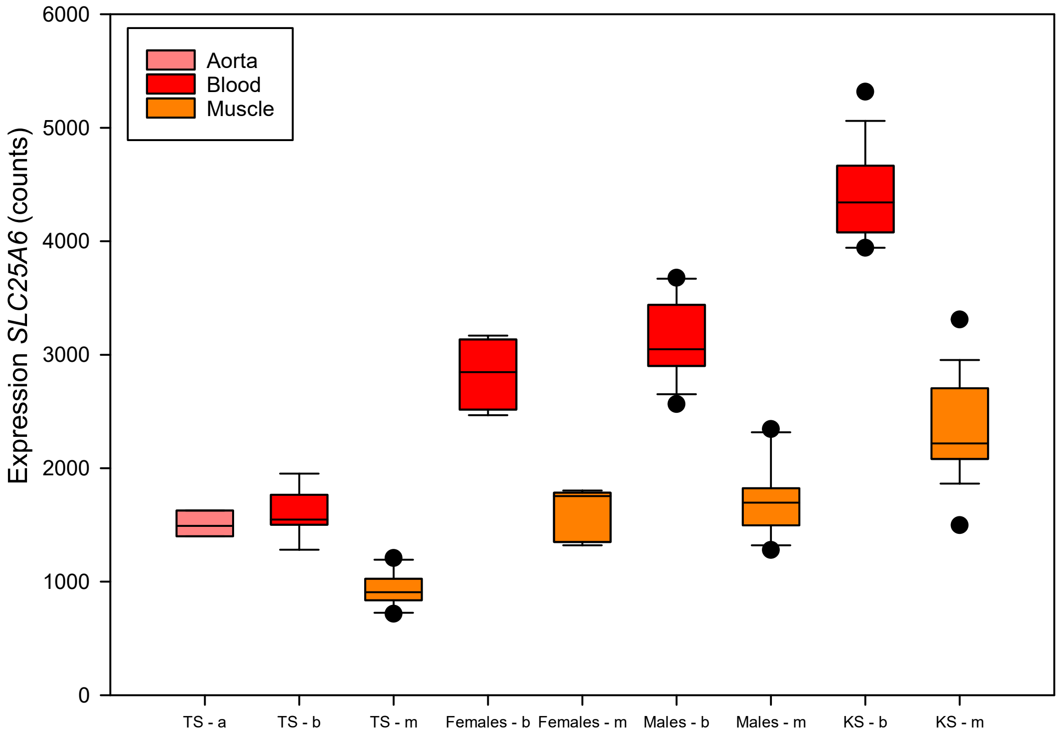

Group	Tissue	Expression of *SLC25A6* (Counts)
**Turner**	Aorta (n=3)	1505.4±114.3
	Blood (n=8)	1594.7±206.8
	Muscle (n=10)	931.2±142.3
**Females**	Blood (n=4)	2832.7±325.3
	Muscle (n=5)	1604.1±234.1
**Males**	Blood (n=16)	3141.7±339.9
	Muscle (n=15)	1715.9±298.2
**Klinefelter**	Blood (n=14)	4406.6±382.1
	Muscle (n=16)	2345.4±432.3

Supplemental Figure 3. Box plot and table showing expression values of *SLC25A6* (counts) in blood (b) and muscle tissue (m) from patients with Turner syndrome (TS), Klinefelter syndrome (KS) and female and male controls from the validation cohort and expression values of *SLC25A6* (counts) in aorta (a) from three patients with Turner syndrome (TS).

A

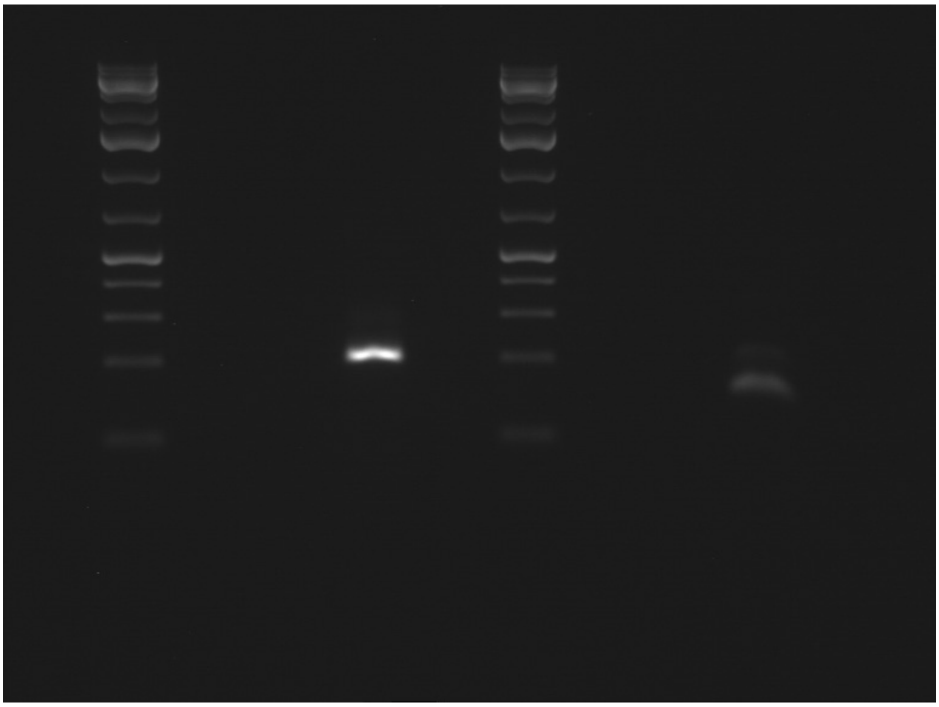

B

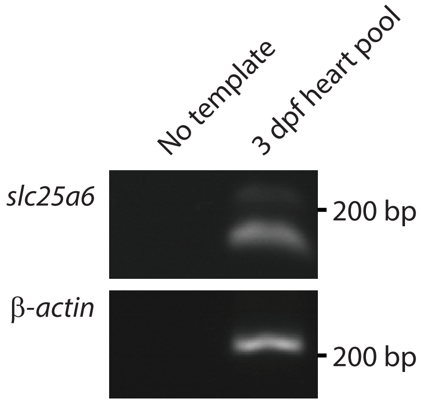

Supplemental Figure 4. Endogenous embryonic cardiac *slc25a6* mRNA is detected by RT-PCR on RNA extracted from a pool of twelve hearts manually microdissected from 3 days post fertilization zebrafish (dpf). (A) Uncropped raw gel image (lane 1 MW, lane 2 empty, lane 3 *β-actin* no template, lane 4 *β-actin* 3 dpf heart pool, lane 5 empty, lane 6 MW, lane 7 empty, lane 8 *slc25a6* no template, lane 9 *slc25a6* 3 dpf heart pool. (B) Cropped gel images of lanes 3 and 4 and lanes 8 and 9.

A

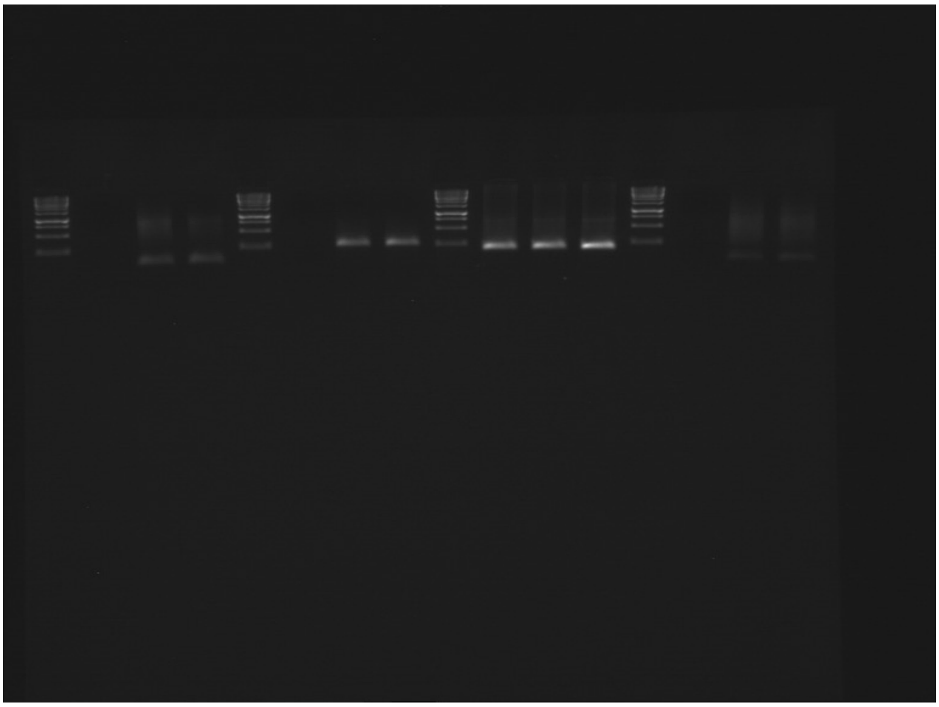

B

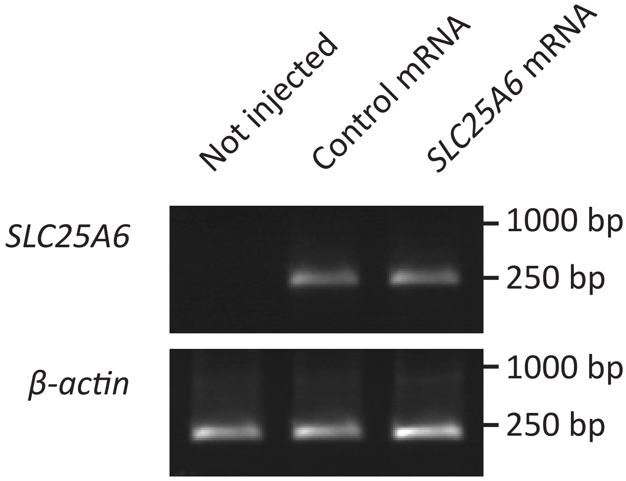

Supplemental Figure 5. Microinjected human wildtype *SLC25A6* mRNA and full length control *SLC25A6* mRNA containing two premature stop codons show similar in vivo stability in zebrafish at 3 days post fertilization. (A) Uncropped raw gel image (lane 1 MW, lane 2 *SLC25A6* primer pair 1 not injected, Lane 3 *SLC25A6* primer pair 1 Control mRNA, lane 4 *SLC25A6* primer pair 1 *SLC25A6* mRNA, lane 5 MW, lane 6 *SLC25A6* primer pair 2 Not injected - selected primer pair, lane 7 *SLC25A6* primer pair 2 Control mRNA - selected primer pair, lane 8 *SLC25A6* primer pair 2 *SLC25A6* mRNA - selected primer pair, lane 9 MW, lane 10 *β-actin* Not injected, lane 11 *β-actin* Control mRNA, lane 12 *β-actin* SLC25A6 mRNA, lane 13 MW, lane 14 *SLC25A6* primer pair 3 Not injected, lane 15 *SLC25A6* primer pair 3 Control mRNA, lane 16 *SLC25A6* primer pair 3 *SLC25A6* mRNA). (B) Cropped gel images of lanes 6-8 and lanes 10-12.

A

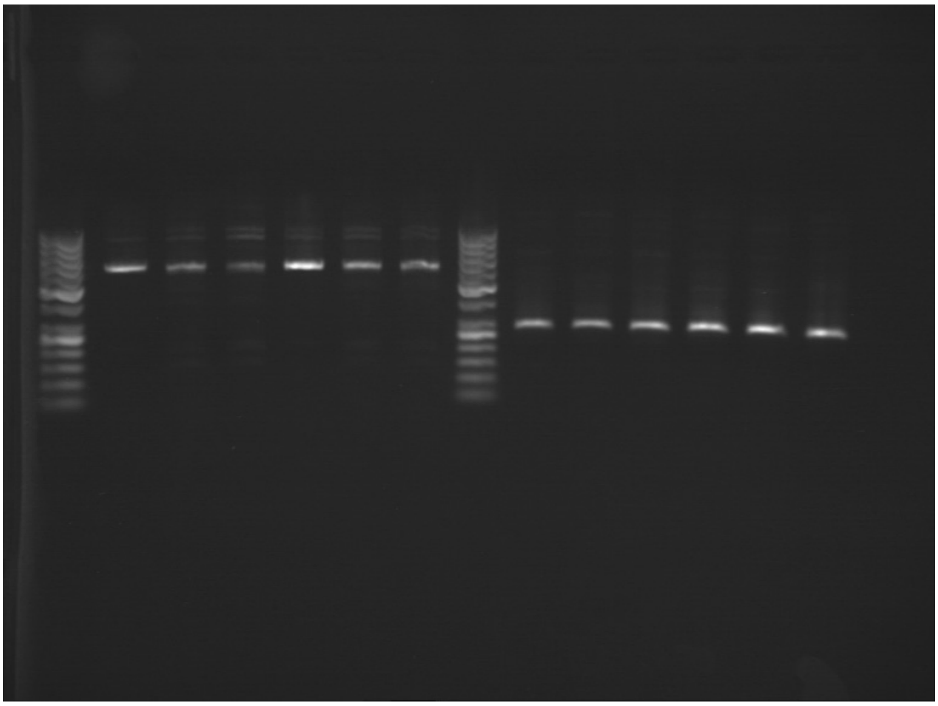

B

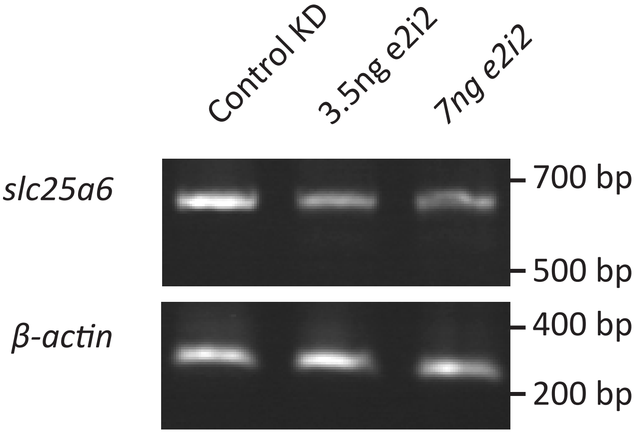

Supplemental Figure 6. Compared to microinjected with control morpholino (Control KD), microinjection of 7ng *slc25a6*-targeted morpholino per embryo resulted in ~ 50% knockdown of endogenous *slc25a6* mRNA levels normalized to *β-actin* at 3 days post fertilization by gel densiometry. (A) Uncropped raw gel image (lane 1 MW, lane 2 *slc25a6* Control KD 2dpf, lane 3 *slc25a6* 3.5ng e2i1 2dpf, lane 4 *slc25a6* 7.0ng e2i1 2dpf, lane 5 *slc25a6* Control KD 3dpf, lane 6 *slc25a6* 3.5ng e2i1 3dpf, lane 7 *slc25a6* 7.0ng e2i1 3dpf, lane 8 MW, lane 9 *β-actin* Control KD 2dpf, lane 10 *β-actin* 3.5ng e2i2 2dpf, lane 11 *β-actin* 7.0ng e2i2 2dpf, lane 12 *β-actin* Control KD 3dpf, lane 13 *β-actin* 3.5ng e2i2 3dpf, lane 14 *β-actin* 7.0ng e2i2 2dpf). (B) Cropped gel images of lanes 5-7 and lanes 12-14.

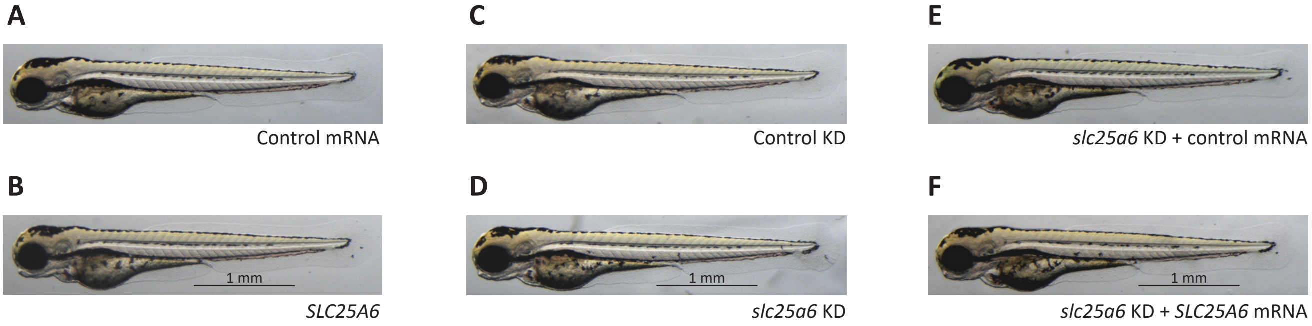

Supplemental Figure 7. Light microscopic images of representative 3 days post fertilization zebrafish embryos (A) overexpressing control mRNA or (B) wildtype *SLC25A6* mRNA indicating no effect of *SLC25A6* upregulation on overall zebrafish embryo morphology. Light microscopic images of representative 3 days post fertilized zebrafish embryos microinjected with (C) control morpholino (Control KD) or (D) *slc25a6*-targeted morpholino (*slc25a6* KD) without coinjection of control mRNA (*slc25a6* KD) or wild type *SLC25A6* mRNA and with coinjection of control mRNA (*slc25a6* KD + control mRNA) or wild type *SLC25A6* mRNA (*slc25a6* KD + *SLC25A6* mRNA) indicating no effect of *slc25a6* knockdown or knockdown rescue on overall zebrafish embryo morphology.

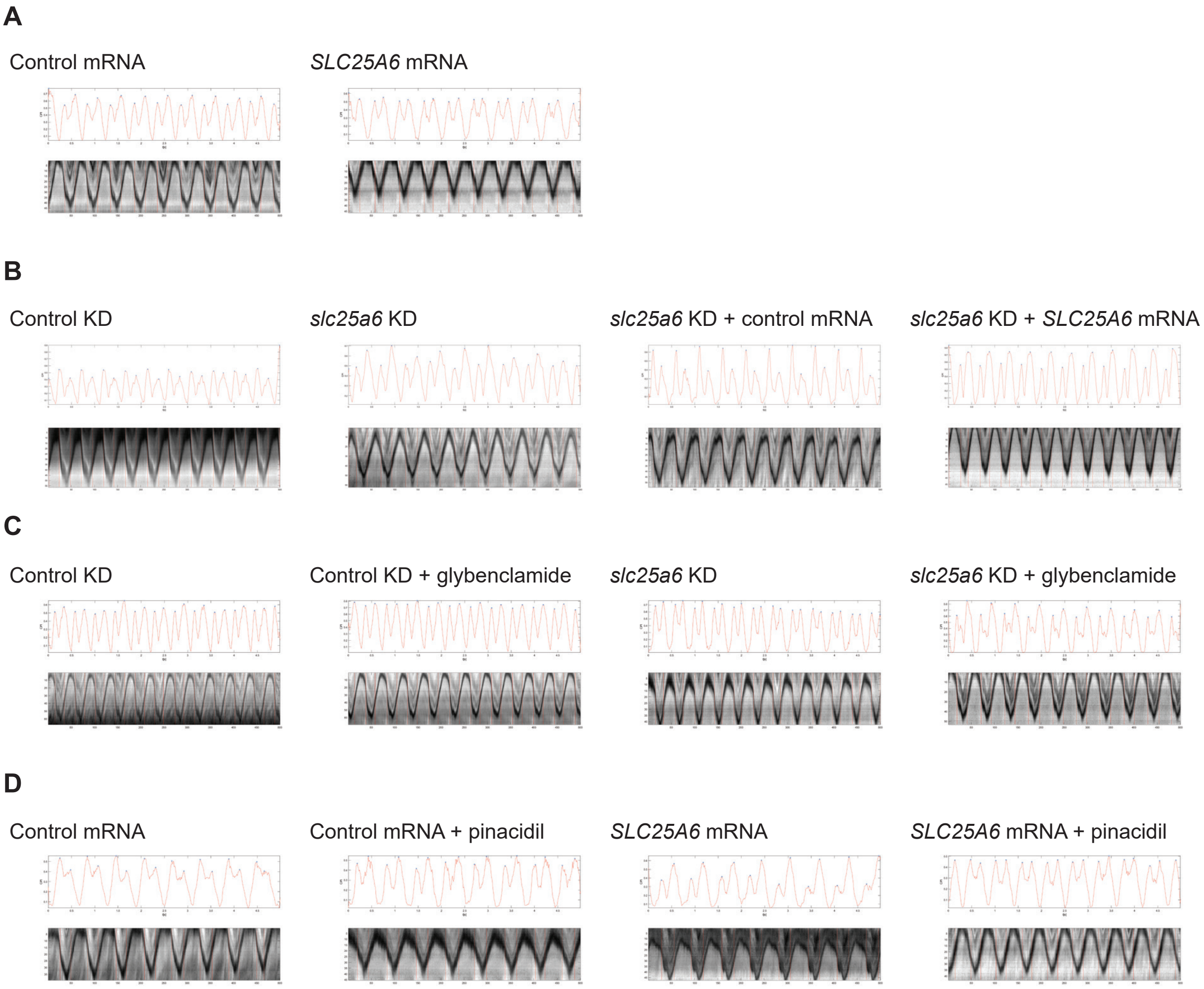

Supplemental Figure 8. Representative output from analysis of cardiac high-speed video recordings. Each video recording was analyzed as previously described[1], resulting in an M-mode displaying the movements of the ventral ventricle wall (lower panels) and the derived changing pixel intensity (CPI) plot (upper panels) permitting quantification of systolic and diastolic interval duration and heart rate. (A) *SLC25A6* overexpression. (B) *slc25a6* knockdown and mRNA rescue. (C) Pharmacological rescue of *slc25a6* knockdown. (D) Pharmacological rescue of *SLC25A6* overexpression.

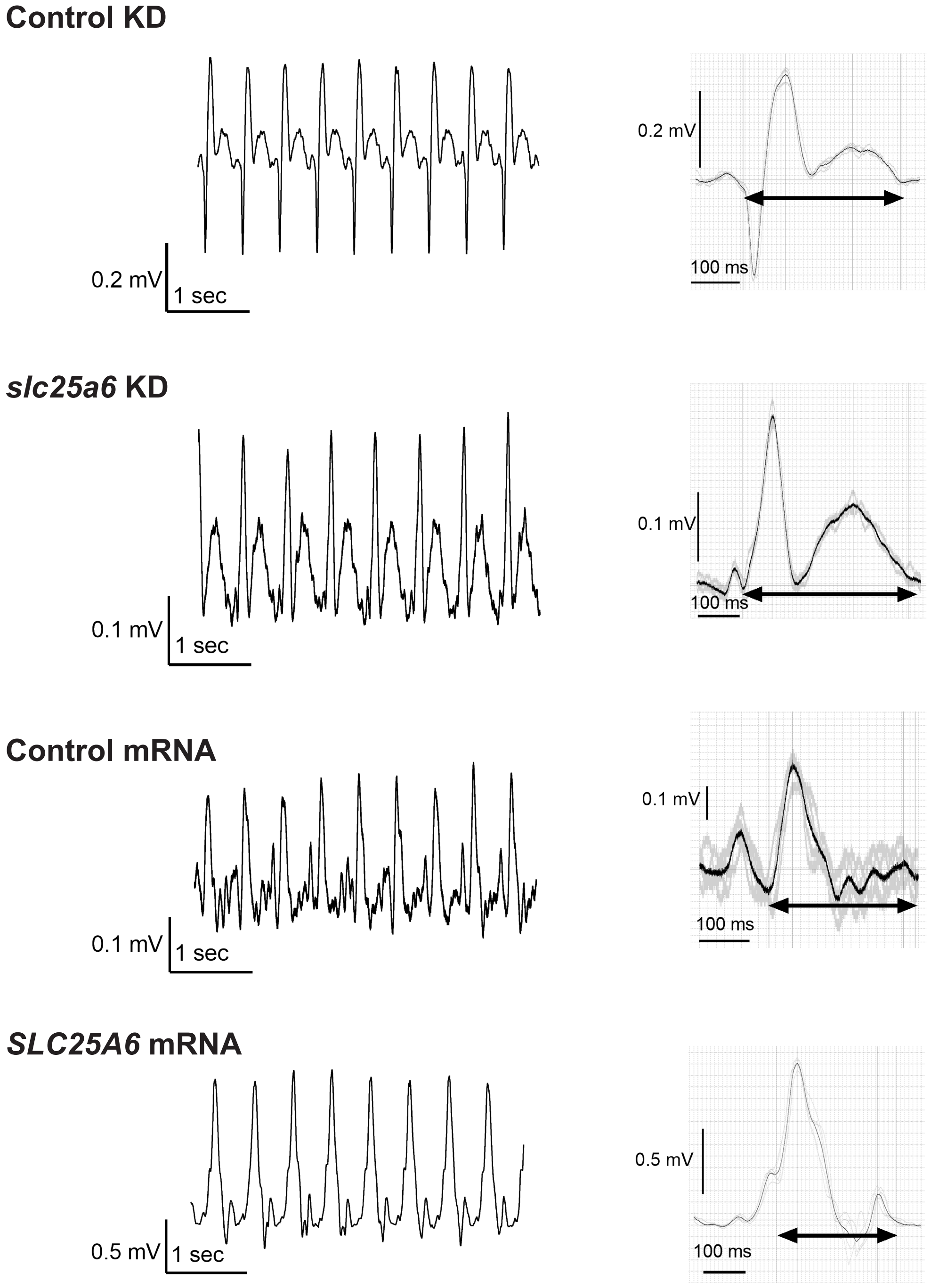

Supplemental Figure 9. Representative ECG recordings from zebrafish embryos with different level of *SLC25A6* expression as indicated. Left panels show the representative traces from embryos controls for *slc25a6* knockdown, *slc25a6* knocked downed embryos, controls for *SLC25A6* overexpression and embryos overexpressing *SLC25A6*. Right panels show the corresponding averaged ECG made in the ECG Analysis Module in LabChart 8 (ADInstruments) as described previously[2]. Shortly, R waves (as the QRS complex maximum) were automatically identified in selected stable registration trace and used for follow-up calculation of R-R intervals and averaging the ECG cycle (right panel). This averaged ECG cycle was used to measure the QTc intervals. See also data in the figure 2.

References

1. Thorsen, K. et al. Loss-of-activity-mutation in the cardiac chloride-bicarbonate exchanger AE3 causes short QT syndrome. *Nat. Commun.* **8**; 10.1038/s41467-017-01630-0 (2017)
2. Christiansen, M.K. et al. Genetic analysis identifies the SLC4A3 anion exchanger as a major gene for short QT syndrome. *Heart Rhythm.* 10.1016/j.hrthm.2023.02.010 (2023)
